# Magnetic particle monitoring on leaves in winter: a pilot study on a highly polluted location in the Po plain (Northern Italy)

**DOI:** 10.1007/s11356-022-20247-5

**Published:** 2022-04-22

**Authors:** Mario Tribaudino, Massimo Solzi, Luciana Mantovani, Patrizia Zaccara, Elisa Groppi

**Affiliations:** 1 Department of Chemistry, Life Sciences and Environmental Sustainability, University of Parma, Parco Area delle Scienze 157/a, Parma, Italy; 2 Department of Mathematical, Physical and Computer Sciences, University of Parma, Parco Area delle Scienze 7/a, Parma, Italy; 3 Present Address: Liceo Scientifico Statale “Gobetti-Segrè”, Via Maria Vittoria, 39 bis, Turin, Italy; 4 Liceo Scientifico “G. Ulivi”, Viale Maria Luigia, 3, Parma, Italy

**Keywords:** Magnetic monitoring, Magnetic susceptibility, SEM–EDS, Bioindicator, Leaves, Particulate matters, Air pollution

## Abstract

Environmental monitoring in Northern Italy, one of the most polluted areas in Europe, is of paramount importance. Leaf monitoring throughout magnetic and scanning electron microscopy (SEM–EDS) analysis could be considered a good complementary analysis to sampling stations, but the lack of evergreen plants in the northern Italy towns may hinder magnetic leaf analysis in the winter season. Therefore, we tested three species of urban vegetation, which are evergreen and commonly found in urban environment, namely *Hedera helix* L., *Parietaria officinalis* L. and *Rubus caesius* L. Magnetic susceptibility, chosen as a simple parameter suitable for monitoring, was measured in seven stations, during the period 25 January 2019 to 8 March 2019 at a weekly step, in the cities of Torino and Parma in the same days. *P. officinalis* and *R. caesius* showed the best response, but also *H. helix* was suitable to detect highly polluted areas. In Torino, the magnetic susceptibility decreased in the last sampling, together with PM10, whereas in Parma it increased, likely for the beginning of the academic period in the University Campus. SEM–EDS analysis was done comparing leaves from the same plant sampled in February 2019, in highly polluted conditions, and in May 2020, after 2 months of very limited traffic, due to national lockdown. Silicate grains of natural minerals, sized between 10 and 20 µm, are present in both samples, whereas Fe oxides, about one micron size, possibly coming from car brake consumption, are prominent in the February 2019 sample. Magnetic susceptibility of leaves form the examined species looks promising to spot urban sites with high metal pollution.

## Introduction

Air pollution is a major factor affecting public health. The WHO in 2019 has estimated 4.2 million premature deaths only in the 2016 year. Airborne particles are among the main atmospheric pollution sources, mostly in urban areas. Particles sized less than 10 µm can penetrate the thoracic region, and, if smaller than 2.5 µm, the alveoli, causing inflammatory effects, respiratory disease, and death as an extreme outcome (Samet and Krewski 2007). It was also suggested in the recent outburst of Covid-19 that atmospheric dust might be a viral carrier, easing the diffusion of the pandemics: higher contagion was found in areas with higher concentration of dust particles (Bontempi 2020; Yao et al. 2020). Although it was questioned whether a direct relation between Covid infection and atmospheric pollution is present, there is no doubt that the constant stress to the respiratory system which affects people living in highly polluted areas makes secondary infections easier.

Environmental pollution from dust is measured in sampling stations with filters that stop all the particles sized less than 10 µm (PM10) or than 2.5 µm (PM2.5). Sampling stations need a fixed location, due to their meter size and the constant need of an electric supply. They are in limited number in a given area, and they are not suitable to perform fine gradient investigation for polluted sites. Also, the bare weight of the deposited PM10 or PM2.5 particles, which is the immediate regulatory information, does not give details either about the composition of the particles, or about the extremely fine sized particles, below 0.1 µm, which can penetrate cellular walls causing systemic disease (Maher 2020). Among the particle components, the metal portion coming from vehicular traffic, albeit limited in weight, has a significant impact on health: trace metals are carried in the particles, which generate free radicals potentially toxic for health (Morris et al. 1995; Maher et al. 2008; Pourret and Hursthouse 2019). Recently, it was shown that metal particles might influence Alzheimer’s disease and mitochondrial dysfunction and cardiac oxidative stress (Maher et al. 2020). Magnetic measurement can be a fast and sensitive proxy to investigate vehicular emissions, metal particles traffic-related and metal rich particulate matter (Winkler et al. 2021). In detail, recent works suggest that brake-wear emissions are the major source of metal-rich airborne particulate pollution in roadside environments (Winkler et al. 2020; Gonet et al. 2021a, b).

Leaves are bio-accumulators of atmospheric particles, among which are magnetic ones. Tree leaves, being a biological tissue, are typically diamagnetic, and for this reason, any magnetic signature comes from the deposited magnetic metals, which essentially originate from anthropic pollution.

Several studies were done on environmental magnetic monitoring (among many others, Cao et al. 2015; Castanheiro et al. 2020). It was found that the magnetic properties depend on the physiology of the leaves, which may, more or less, catch and retain the particles (Moreno et al. 2003; Hofman et al. 2017).

For example, in Italy environmental studies were previously done using *Quercus ilex* L. leaves in Rome (Fusaro et al. 2021), or *Tilia cordata* Mill. in Parma, northern Italy (Mantovani et al. 2018).

*Q. ilex* has sticky leaves that are efficient for immobilizing PM, being suitable for biomonitoring studies (Muhammad et al. 2019); unfortunately, it is a Mediterranean species, absent in the continental climates of the Po plain. In *T. cordata*, the work of Mantovani et al. (2018) found a significant correlation between magnetic properties of the leaves and those of PM10 filters, averaging the data collected in several days. The sampling of *T. cordata* was done between July and mid-November 2017, up to the leaf fall. Leaf fall affected the potential in the area of the Po plain as a monitoring proxy of *T. cordata*, which missed the highest pollution peak reported by the magnetic analysis of the PM10 filters, performed till the end of December 2017 (Mantovani et al. 2018).

The above investigation highlighted that, in the application of magnetic analysis in leaves, we have to take care of two important points: (a) in areas with higher pollution in winter, like the Po plain, leaf fall hinders winter sampling; and (b) the features of the leaf page are strictly connected to the efficient trapping of magnetic particles. In a recent work that compares 96 plant species, Muhammad et al. (2019) found that in a plant with sticky leaves, like *Q*. *ilex*, there is a higher concentration of particles than in a plant with smooth leaves, like *T. cordata*.

When trees with leaves suitable to collect magnetic particles are absent in a given area, a possible alternative may be evergreen weed or ivy. They are commonly found in towns, often at the roadside, and do not need special care in sampling, and their distribution is widespread. However, special care has to be paid in choosing the species. Plants which are found only in gardens, like *Osmanthus fragrans* Lour. and *Ligustrum lucidum* W. T. Aiton (Dai et al. 2020), are of little use in sampling at traffic conditions, and so is the potentially interesting roof green, which is absent in most towns (Speak et al. 2012). An exception is ivy (*H. helix*), which was the subject of two recent papers (Castanheiro et al. 2016, 2020). In the first work (2016), ivy was successfully proposed for magnetic monitoring, showing strong differences between forest, rural and industrial sampling sites. SEM–EDS analysis on many particles showed a higher iron particle concentration in ivy sampled from industrial areas. In that work, however, an assessment on the Fe-bearing silicate mineral dust of natural respect to anthropic origin was not done. In the 2020 paper of Castanheiro et al., further sampling was not done, and it was shown that metal Fe is leachable. The magnetic behaviour of *H. helix* was also reported by Muhammad et al. (2019). Incidentally, Muhammad et al. (2019) reported just 5 evergreen broadleaves and two climbers, among which is *H. helix*, out of 96 plants.

In this work, we assessed suitable species for magnetic pollution monitoring in wintertime in the Po plain region. Tests were done in Parma in a suburban-countryside area within the University Campus and in Torino, a near highly polluted roadside. We examined three common evergreen plants, namely *H. helix*, *P. officinalis* and *R. caesius*. The chosen species are common in urban conditions; they were selected after checking their presence in the town alleys and roads. They keep leaves also in winter season, and show a good response for magnetic monitoring (Castanheiro et al. 2016). Moreover, they are at the road level, and can be easily sampled. *P. officinalis* takes its name from the Latin word *paries* (wall): it grows in cracks on the walls, with very little soil; it can provide an environmental marker in places where other urban vegetation is absent, and it is widespread even in central towns. *H. helix* is found with hedges or trees as a widespread invasive host. *R. caesius* is instead most found in suburban sites, and in public parks. *P. officinalis* and *H. helix* were sampled in Torino, *R. caesius* and *H. helix* in Parma.

The ultimate goal of this research is to provide an easy, fast and cheap proxy for magnetic pollution readily available to map polluters at a fine scale. For this reason, in-depth magnetic mineralogy analyses were not carried out, but only magnetic susceptibility was assessed as a marker to measure for repeated samples (Petrovský et al. 2000; Gautam et al. 2005; Szönyi et al. 2008). In particular, many literature data demonstrated that it is a fast and accurate proxy of the traffic-related vehicular emission (see for example Winkler et al. 2021). The intensity of low-field magnetic susceptibility mostly depends on the concentration of ferrimagnetic minerals (such as magnetite and maghemite) derived from anthropogenic traffic-related and industrial pollution (Morris et al. 1995; Szönyi et al. 2008). In principle, magnetic susceptibility is related not only to concentration but also to the average grain size and the average bulk composition of the ferrimagnetic minerals. It corresponds to the contribution of multi-domain, stable single-domain and superparamagnetic particles. Complementary techniques are required to determine the relative contributions of grain size and compositional changes, in particular SEM–EDS and hysteresis properties. In this work, in addition to magnetic susceptibility, SEM–EDS investigation was done on few selected leaves among *P. officinalis* and *H. helix* sampled to better understand the distribution, size and composition of the deposited particles of the examined species. It should be noted that the work was carried out in collaboration with high school students of Parma and Torino, (PCTO project) trying to sensitize the new generations to particulate matter air pollution.

## Material and methods

### Sample collection

The sampling was done in Torino, in few sites at the centre of the town, and in Parma, in the University campus, at the outskirts of the city. The two towns are located in the Po plain, an area showing climatic, geographic and environmental circumstances that result in heavy PM10 burden.

**Fig. 1:**
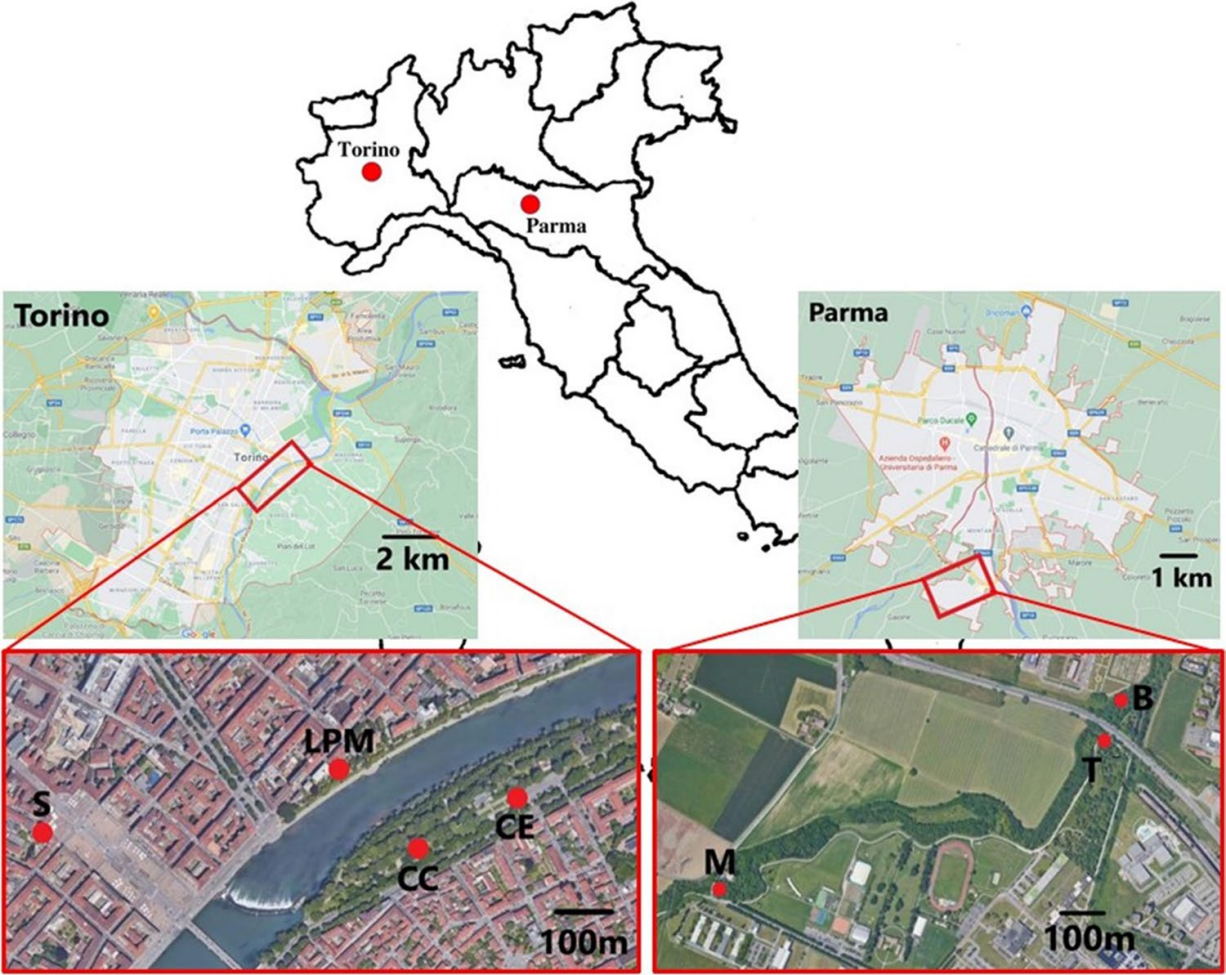
Map position and of the sampling site PR: Parma, TO: Torino, B: Boschetto, T: tangenziale, M: Montanara, S: school, LPM: Lungo Po Macchiavelli, CE: ceppo, CC: Corso Casale

The town of Torino is one of the most polluted in Europe, with an average daily PM10 exposure above 40 µg/m^3^ and with little below 100 days exceeding the 50-µg/m^3^ limit (De Donno et al. 2018). In the same town, however, due to the presence of the river Po and of the hillside, areas with lower pollution coexist at little distance from the more polluted ones. Four sites in Torino were chosen a few hundred meters apart, representing different urban environments. Two sites were near a highly trafficked road, Corso Casale (Corso Casale—CC and Ceppo—CE), one at the car entrance of the Gobetti-Segrè secondary school, in the historical centre of Torino (School—S), and the last along a pedestrian way bordering the river Po (Lungo Po Machiavelli—LPM), just on the other side of the river with respect to the sampling sites in CC (Fig. 1). In the CE sampling site, we sampled *H. helix* on a bush at the side of the road, in the CC site, at about 200 m from CE, and in LPM we sampled *P. officinalis* and *H. helix* below a hedge; at the entrance of the school (S), only *P. officinalis* was taken.

The town of Parma shares a similar atmospheric condition, with long-lasting winter high pressure and peaks of pollution. *R. caesius* and *H. helix* were sampled in three sites in Parma: one was located near an urban highway (Tangenziale—T), but off from the centre town, another at a car exit of the campus (Strada Montanara—M), and the last one in a woodland at about 50 m from the urban highway, along a bike trail (Boschetto –B), near the University Campus (Fig. 1).

A high traffic load is present in Torino CC and in the urban highway in Parma (T). In Torino, the traffic is more of the stop and go type, with traffic lights, and cars queuing in the morning, whereas in Parma little use of brakes and gasoline exhaustion is expected from passing cars. The coordinates of the sampling sites site are reported in Table 1.

**Table 1:** Summary table of the sampling site with GPS coordinates and sampling species with some specification: PR: Parma, TO: Torino, B: Boschetto, T: tangenziale, M: Montanara, S: school, LPM: Lungo Po Macchiavelli, CE: ceppo, CC: Corso Casale, H: *H. helix*, R: *R. caesius*, P: *P. officinalis*, U: up, D: down, Y: young, O: old

Sample site	GPS	Sampled species	Specification	Area description
PR-B	44° 46′ 17.4″ N 10° 19′ 02.3″ E	R, H	U, D	50 m from the urban highway, along a bike trail
PR-T	44° 46′ 15.5″ N 10° 18′ 58.3″ E	R,H	U, D	Near an urban highway
PR-M	44° 46′ 02.3″ N 10° 18′ 14.2″ E	R, H	U, D	Car exit of the University campus
TO-S	45° 03′ 55.0″ N 7° 41′ 34.7″ E	P		High school car entrance
TO-LPM	45° 03′ 59.8″ N 7° 42′ 02.8″ E	P, H	O, Y, U, D (only for H)	Along a pedestrian way bordering the river Po
TO-CE	45° 03′ 56.5″ N 7° 42′ 13.4″ E	H	O, Y	Near a highly trafficked road
TO-CC	45° 03′ 53.0″ N 7° 42′ 05.7″ E	P,H	O, Y (only for H)	Near a highly trafficked road
TO-CC	45° 03′ 53.0″ N 7° 42′ 05.7″ E	P, H		Sampled in pandemic lockdown (12 May 2020)
PR-PIEVE	45° 01′ 36.1″ N 10° 08′ 08.3″ E	R,H		Middle of the woods, near river Po, 40 km from Parma
TO-PDM	45° 18′ 27.5″ N 7° 29′ 28.5″ E	H		Mountainside, 50 km from Torino

In Parma, in all the stations leaves were sampled twice, one at road level, the other at the top of the plant, between 1 and 2 m, and named as down (D) and up (U), respectively.

In Torino, also young and old leaves of *H. helix* were sampled separately to verify a possible accumulation effect; we labelled young (Y) the light green leaves stemming from the shoots and old (O) those darker green. Moreover, in the LPM, where *H. helix* was present down close to the walkway and above in a terrace at two meters from the walkway, the leaves down and above the terrace were sampled separately. As a result, in LPM four specimen were taken for each sampling day, up (U), down (D), old (O) and young (Y), and except *P. officinalis*, in the other stations two samples were taken, up (U) and down (D).

The sampling was performed once a week and at the same date in Parma and Torino, over 7 weeks between 25 January 2019 and 8 March 2019. In Torino, the sampling of March 1st was missed. For each sample, 3 leaves were taken. In all, but one day (1 February 2019), the weather was fine, sunny or little cloudy, with little wind. On February 1, we had snow mixed with rain in Torino, and rain in Parma. In Parma, the sampling was done between the winter and spring academic periods, except for the last sampling on March 8th, at the starting of the academic lessons in the University Campus, which involved significantly higher car traffic.

In Torino, during the months of January and February 2019 the limit of 50 μg/m^3^ was overtaken in 54 out of 58 measured days, whereas in March it was always below 50 μg/m^3^. During the sampling period, PM10 for 8 days was over 100 μg/m^3^ with a top at 136 μg/m^3^. In 4 out of 6 sampling days, PM10 was higher than 80 μg/m^3^, with maximum at 122 μg/m^3^ on 8th February. In Parma, PM10 was slightly lower but still beyond critical limits.

Another sampling of *P. officinalis* and *H. helix* was repeated in the two highest polluted stations along the CC, in Torino, on 12 May 2020. The sampling is notable as it was done after the 2 months, March–April, of strict lockdown during the Covid-19 first pandemic rise. Traffic was almost absent during lockdown, and still lower than before the lockdown on 12 May 2020. On the same day, the measured PM10 in Torino was 16 µg/m^3^.

Together with the sampling site, we examined two reference samples for *H. helix*, located far from any pollution source, one near Pieveottoville on the banks of the river Po, at about 40 km from Parma, and the other at mountainside, at Pilone del Merlo, about 50 km from Torino. In the Pieveottoville station, also *R. caesius* was sampled as reference sample. In both cases, the sampling was done in January 2019. The samples taken as reference come from two sites without any nearby sources of pollution or trafficked roads.

### Sample preparation and magnetic susceptibility

After each sampling, three leaves were collected and dried in oven at 50 °C for 2 days. Then, the leaves were crushed in a ceramic mortar, discarding the veins and petiole. The crushed leaves were put in a 0.2-ml gel capsule and then weighted. The typical mass of pulverized leaves in the capsule was in the range 80–140 mg. The alternative current (AC) mass susceptibility was measured at the Department of Mathematical, Physical and Computer Sciences of the University of Parma by means of the Oxford Instruments MagLab System 2000 platform equipped with the Magnetic Measurement Probe. The characteristics of this instrument are AC magnetic field frequency in the range 10 Hz–10 kHz, maximum RMS (root mean square) amplitude 4 kA/m and estimated sensitivity, in terms of mass susceptibility, $\pm 2.5\times {10}^{-10}\frac{{\mathrm{m}}^{3}}{\mathrm{kg}}$ at 4 kHz. The measurements were performed at either 998 Hz or 3998 Hz with an AC field of 1.6 kA/m and repeated at least 15 times each. The background signal due to the capsule and to the sample holder was measured and subtracted. In all samples, the resultant net susceptibility was significantly beyond the instrument sensitivity. Contrarily to what we found in Castanheiro et al. (2020), in no case negative susceptibility was observed. A total of 150 samples were measured for this study.

### SEM–EDS analysis

SEM–EDS analyses were performed on *Hedera* and *P. officinalis* sampled in the CE and CC, on 8 February 2019 and 12 May 2020. These data were chosen as indicative of opposite environmental conditions: on 8 February 2019, the highest PM10 and susceptibility were observed (103 μg/m^3^ and 42 * 10^−8^ m^3^/kg, respectively). On May 12th instead, after lockdown and in the environmentally more favourable spring weather, we had the lowest PM10 and measured susceptibility (16 μg/m^3^ and 4.9 * 10^−8^ m^3^/kg). Moreover, a piece of *R. caesius* from the Pieveottoville reference site was analysed, as representative of sample from a non-polluted area (susceptibility value 0.72 * 10^−8^ m^3^/kg). The leaves were in pieces smaller than 1 mm.

The analyses were done with a Jeol 6400 Scanning Electron Microscope equipped with an Oxford EDS (energy-dispersive system) microprobe. Microprobe analysis operating conditions were 20 kV and 1.2 mA current, ~ 1-mm beam diameter and 60 s counting time; several analytical points and chemical maps per sample were done. SEM images were obtained using both back-scattered and secondary electron detector, to assess the presence of different phases, as well as their morphology and composition. The instrument resolution does not allow identifying particles with a dimensional size less than 0.5 µm. In few images, a further analysis was done to determine the particle distribution and size. The software ImageJ2 (Rueden et al. 2017) was used, to encircle mineral particles from secondary electron images, and then to select grains smaller than 10 and 2.5 µm. The number of particles, their area and percentage coverage were obtained. The same analysis was done from compositional maps, to assess the area where for a given element we have a significant emission.

## Results and discussion

### Magnetic properties of different plants

**Fig. 2:**
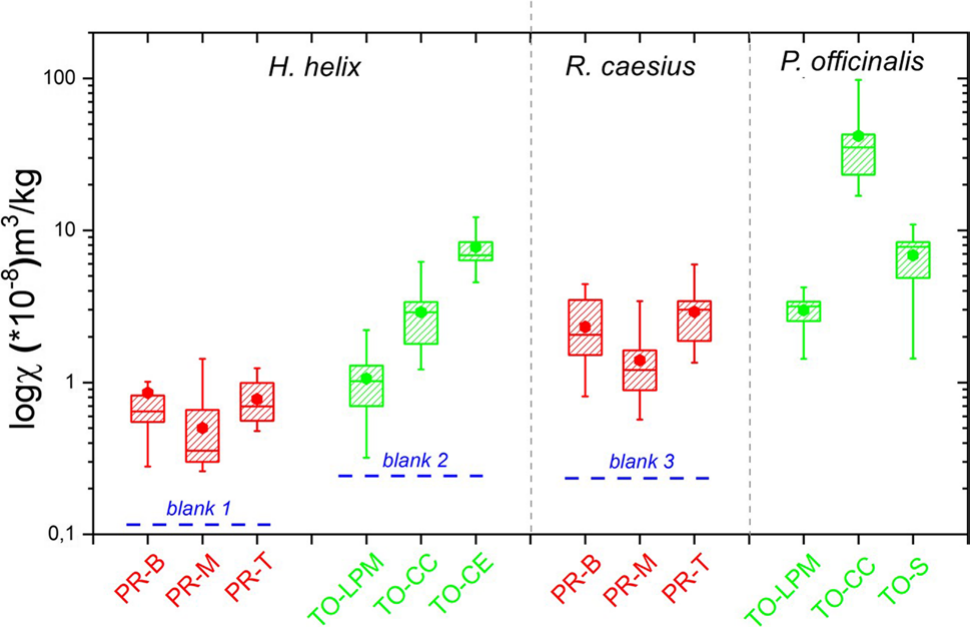
Box plots of the susceptibility values (in log scale) for *H. helix*, *P. officinalis* and *R. caesius* of Torino (TO in green) and Parma (PR in red) sampling. The filled circle represents the mean value. PR: Parma, TO: Torino, B: Boschetto, T: tangenziale, M: Montanara, S: school, LPM: Lungo Po Macchiavelli, CE: ceppo, CC: Corso Casale. The blue line represent the value in the three reference samples (see Table 1 for details)

A box plot showing for each sampling site and for the entire collection period the distribution of the magnetic susceptibility for *H. helix*, *R. caesius* and *P. officinalis* in Parma and Torino is shown in Fig. 2. In Table 2, the 1-way ANOVA test is reported.

**Table 2:** One-way ANOVA used to test for differences species and sampling locations. For the ANOVA test, the significance value is < 0.05. PR: Parma, TO: Torino, B: Boschetto, T: tangenziale, M: Montanara, S: school, LPM: Lungo Po Macchiavelli, CE: ceppo, CC: Corso Casale, H: *H. helix*, R: *R. caesius*, P: *P. officinalis*

	(H) PR-B	(H) PR-M	(H) PR-T	(H) TO-LPM	(H) TO-CC	(H) TO-CE	(R) PR-B	(R) PR-M	(R) PR-T	(P) TO-LPM	(P) TO-CC	(P) TO-S
(H) PR-B	*	0.06603	0.80907	0.24148	0.00003	0.00001	0.00025	0.04880	0.00001	0.12794	0.00026	0.00000
(H) PR-M		*	0.15995	0.00026	0.00000	0.00000	0.00001	0.00046	0.00000	0.12764	0.00024	0.00000
(H) PR-T			*	0.23690	0.00866	0.01071	0.01892	0.13865	0.00405	0.44443	0.05170	0.00678
(H) TO-LPM				*	0.00000	0.00000	0.00003	0.09848	0.00000	0.04721	0.00000	0.00000
(H) TO-CC					*	0.00201	0.25111	0.00220	0.97236	0.16332	0.00119	0.00198
(H) TO-CE						*	0.00027	0.00006	0.00091	0.18850	0.00605	0.67912
(R) PR-B							*	0.02259	0.20022	0.12918	0.00039	0.00018
(R) PR-M								*	0.00086	0.14410	0.00050	0.00002
(R) PR-T									*	0.12969	0.00046	0.00085
(P) TO-LPM										*	0.38224	0.34506
(P) TO-CC											*	0.03793
(P) TO-S												*

The different bioaccumulation when exposed to the same environmental pollution could be investigated in the cities where two species were sampled together: LPM and CC for *H. helix* and *P. officinalis* and the three stations in Parma for *H. helix* and *R. caesius*. In these stations, *H. helix* has a significantly lower response than the companion species (Fig. 2). *R. caesius* and *P. officinalis* were not compared in a single station, but in the station LPM (Torino) and T (Parma) *H. helix* shows similar susceptibility, giving a benchmark for comparing together the companion species, *P. officinalis* in Torino and *R. caesius* in Parma. As *R. caesius*, in Parma, and *P. officinalis*, in Torino, show similar susceptibility values, we may assume that the bioaccumulation of magnetic particles is similar. To note, this is an attempt to evaluate among the three species, which one has a better magnetic response without any comparison, among the different species.

The highest susceptibility for *P. officinalis* was found in Torino, in CC, at about 10 m from the roadside. A lower value was observed in the S station, which is at the car entrance of the school court.

Although in both stations we are at a roadside, the difference may be explained by the traffic blockage between 7.30 and 10.00 in the central zone of the town, where the school station is located. The same blockage deviates most of the traffic in the CC, with queues at the traffic lights. The lowest susceptibility for *P. officinalis* was found in LPM, a road at the banks of the river Po, about 150 m from CC, with the river in between, closed to vehicular traffic and likely shielded by other pollution sources by tall buildings on the back.

A gradient from the roadside was observed with in Torino: the highest value was found in the plant at the border of the road (CE), a lesser one in *Hedera* in the CC station (same station as *P. officinalis*), and the lowest, but still significant value in the LPM station. To note, the value observed for the LPM station is comparable to that found near the urban highway in Parma for the *H. helix*, suggesting an aerosol dispersion for traffic in the sub-urban site.

In Parma, *R. caesius* shows for any station higher magnetic susceptibility than *H. helix*. Comparing the different stations in Parma, *H. helix* and *R. caesius* show higher susceptibility in the B and T stations, than in the M one. This was somewhat surprising, as the M station is on the roadside of a secondary exit of the University campus, but the lower magnetic susceptibility value may be referred to the lower vehicular traffic, as sampling was performed during an academic hiatus.

**Fig. 3:**
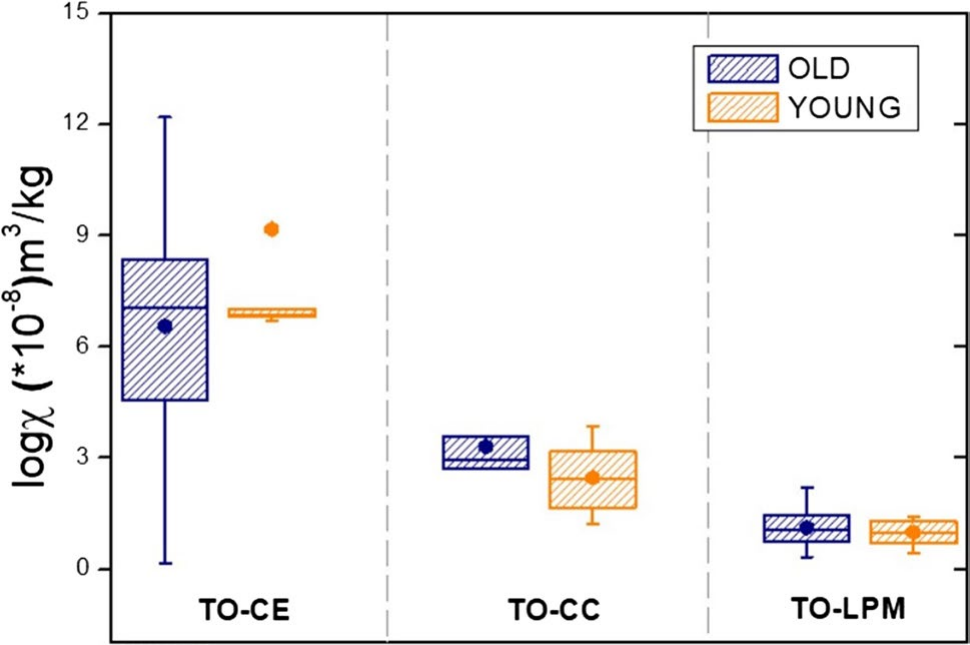
Box plots of the distribution values in function of the old and young leaves of *H. helix*. The choice between young and old leaves was made in the Torino sites Ceppo, Casale and Lungo Po Macchiavelli

In *H. helix*, the new-born leaves, recognized by their lighter green aspect, and by the proximity to the apex of the vine, were sampled separately to the older ones, showing a dark green aspect. As shown in Fig. 3, in none of the three sampling sites in Torino did we observe any difference between old and young leaves (*p* = 0.3, 0.30, 0.48, respectively in CE, CC and LPM). Also, the difference between leaves sampled up and down was not significant (*p* > 0.05).

### Leaf magnetism and environment

**Fig. 4:**
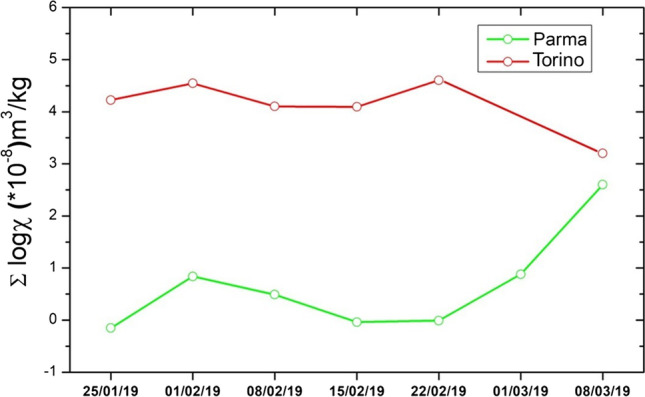
Sum of the logarithm susceptibility of all the measurements done on the species analyzed in Torino and Parma

Figure 4 reports the sum of the logarithm susceptibility in function of the sampled days. The sum of the log is used to average the results with different values range. The final value reported in the graph include all the measurements done, which is the measure on *H. helix* and *P. officinalis* for Torino or *H. helix* and *R. caesius* for Parma, including old and young, up and down measurement.

We observe an inverse trend in the last day in Parma and Torino, respectively: Parma shows an increase in magnetic value whereas a decrease is observed in Torino. In Parma, as previously discussed, this is likely related to the beginning of the academic period, with higher traffic burden in the University Campus. In Torino, the decrease is motivated by the reduced PM10 emission, due to the breakup of the high-pressure winter regime, with windy and sunny days. Also, no difference is found between the February 1st sampling, done with heavy rain mixed with snow, and the others in February, indicating that the washout effect is not effective in the investigated species.

**Fig. 5:**
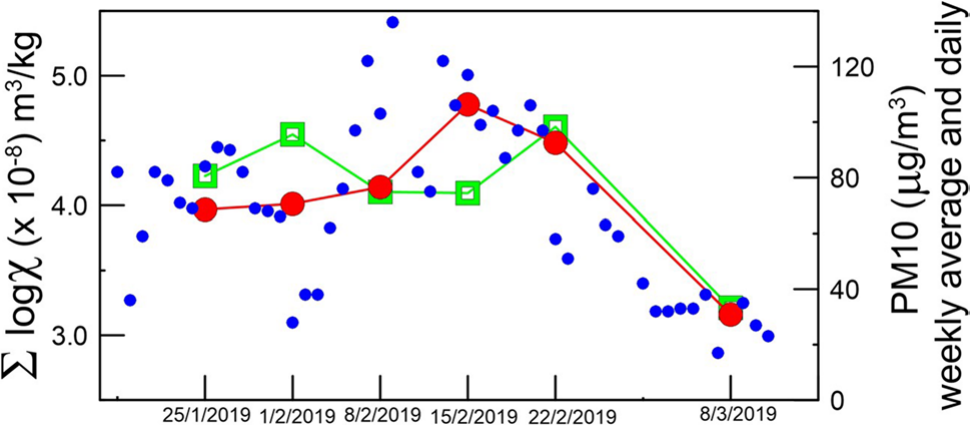
Comparison between the magnetic susceptibility given by the sum of the logarithm susceptibility and the PM10 value from ARPA measurement. Red line shows weekly averaged PM10. Since the comparison is made with the Rebaudengo PM10 station which is a station located in a polluted area, the comparison is made with the sampling of the polluted areas, i.e. CC, CE and S

A relation is found with PM10 measured in the Rebaudengo PM10 sampling station in Torino, comparing day-by-day PM10 data with magnetic susceptibility. The Rebaudengo PM10 station is located in a highly trafficked environment, similar to that of the studied stations. The PM10 day-by-day changes significantly with atmospheric pressure, wind and rain, although retaining values were well over regulatory limits. Notably, it was lower on the second day of sampling, due to snow. On a weekly average, the PM10 values stand on a more stable trend, with a decrease at the beginning of March (Fig. 5). We can consider leaf magnetic monitoring as an average indication of more than 1 day not strictly related with PM10.

All the samples show higher susceptibility than the reference samples. The reference samples showed however a significant value, which could be a background value in the Po plain.

The two May 2020 samples show lower magnetic susceptibility value than the corresponding measures done during the winter 2019 sampling. This likely occurred for the lower but present vehicular traffic after lockdown.

### Electron microscopy

**Fig. 6:**
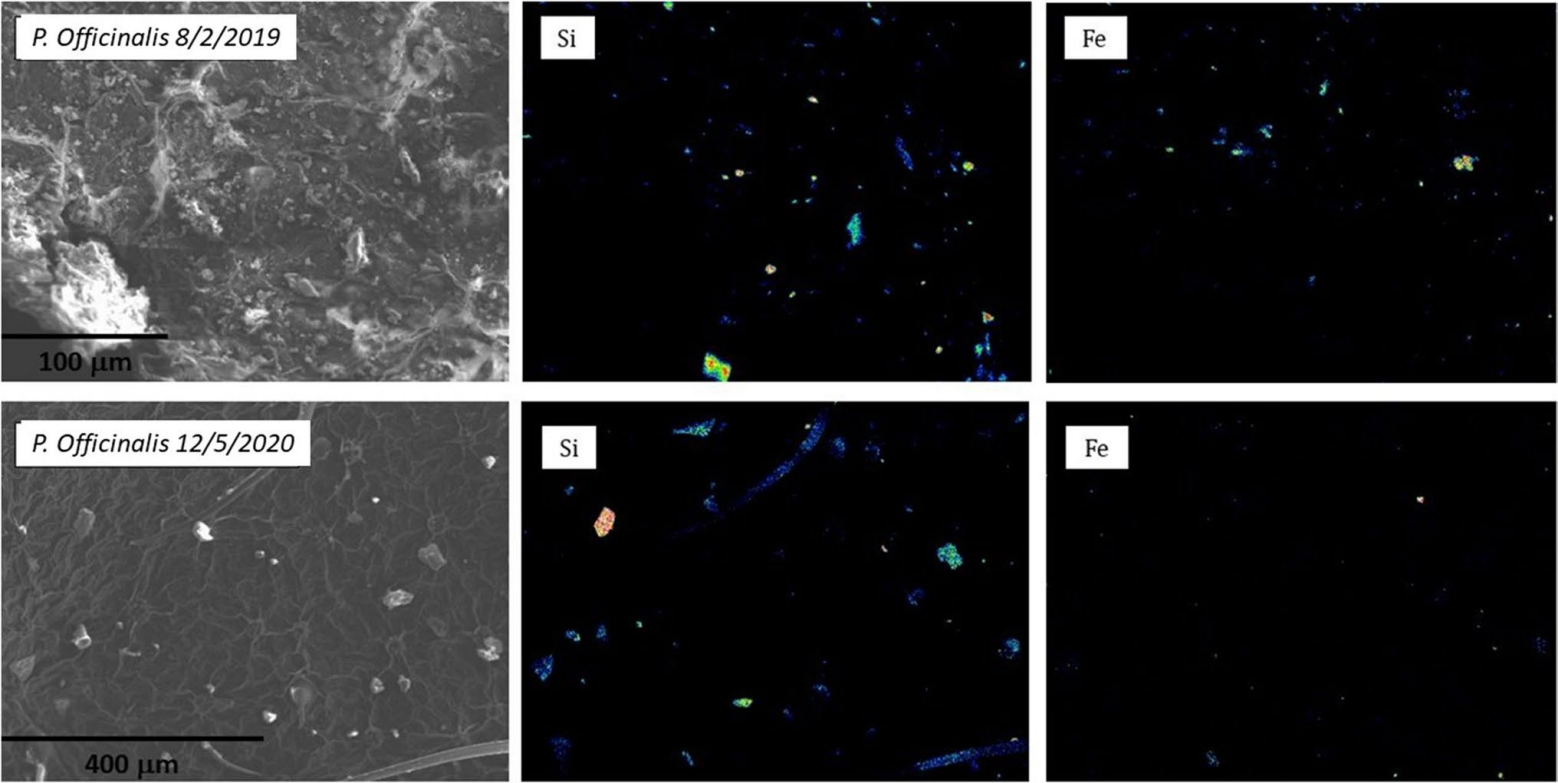
Secondary electron image and maps of Si and Fe before (above) and after (below) lockdown in *P. officinalis* Corso Casale

In Fig. 6, we show secondary electron images of *P. officinalis* collected on 8 February 2019, during sampling for magnetic monitoring, in normal traffic conditions, and in a leaf from the same plant on 12 May 2020, just after the March–April 2020 lockdown, and reduced traffic. Mineral dust, from natural or anthropic sources, is apparent; areal analysis showed the percentage deposition and the relative proportion of different grain sizes. The same analysis was repeated on the blank *R. caesius* sample.

**Fig. 7:**
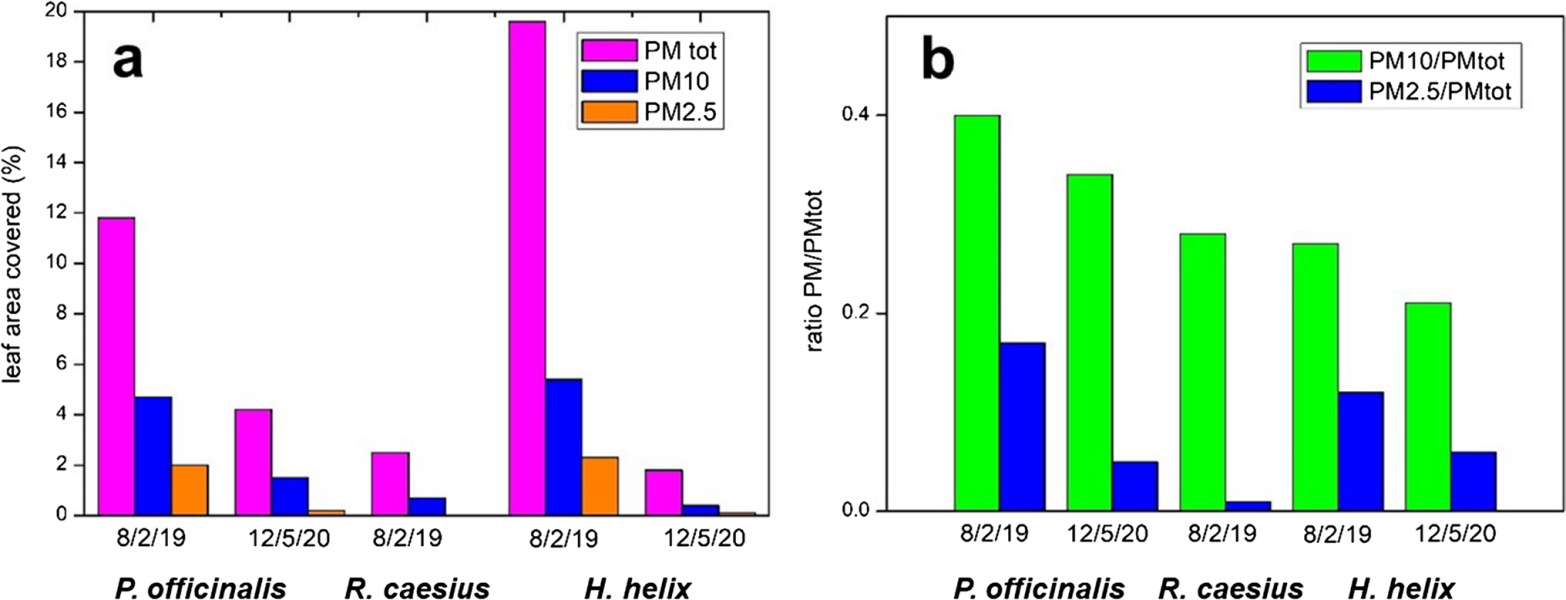
**a** Percentage of the area covered by mineral grains (PMtotal, PM10 and PM2.5) obtained by the SEM image. **b** Ratios between percentages are of grains larger than 10 and 2.5 µm with total grains

**Fig. 8:**
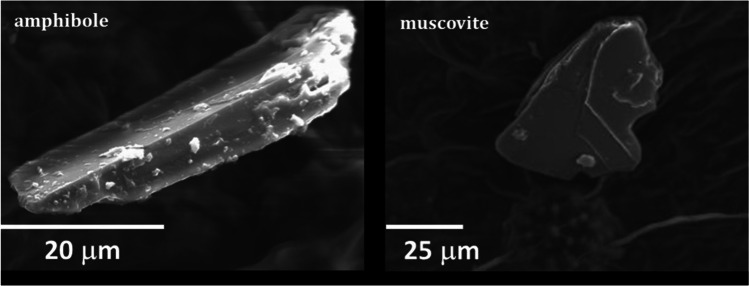
Crustal silicates in the post-lockdown *P. officinalis*

In the *P. officinalis* leaves on 8 February 2019, the dust deposited is more abundant than in May 2020, for any grain size. In Fig. 7a, the area of the SEM image covered by the particles is measured together with the dimension of the particulate matter and the percentage is reported. Then, an estimation of the ratio between the PM10/PMtotal and PM2.5/PMtotal in the sample of 2019 and 2010 are represented. A decrease in all the particles, especially those smaller than 2.5 micron, is clearly visible after the lockdown. SEM–EDS analysis shows that most of the larger grains are mineral silicates: prevailing phases are quartz and a range of phyllosilicates broadly ascribed to the clay mineral family, among which illite and chlorite are recognized. Other silicates are also present, such as amphibole, albite and antigorite, as well as carbonates (calcite and dolomite). Figure 8 shows mineral grains respectively of tremolitic amphibole and muscovite mica with a crystal chemical composition K_0.04_(Ca_1.79_Na_0.21_)Mg_2.86_Fe_1.39_Al_0.65_Si_8_O_22_(OH)_2_ and K_2.1_(Al_2.66_Mg_0.38_Fe_0.92_)Al_1.07_Si_6.93_(OH)_4_ respectively. The same minerals were found in a previous investigation on mineral dust (e.g. Lu et al 2007), except for amphibole and antigorite. The occurrence of the latter can be explained by the many outcrops of ophiolite in western Alps (i.e. the Lanzo Massif) and/or with the large use of these rocks as ornamental stones, railway ballast and road asphalt in the town of Torino (Debret et al. 2013).

The above phases show almost no ferrimagnetic properties. Particles with composition corresponding to magnetic minerals were found as tiny grains of magnetite and Fe–Mn alloy. EDS analysis of magnetite was done in few grains larger than 1 µm, showing a significant content in Cr, Ti and Zn (0.5, 0.6 and 3.0 wt%, respectively).

The distribution of the Fe-rich phases can be assessed by EDS maps of the Fe content. Fe is not by itself an indicator of ferrimagnetism: Fe is present in several diamagnetic minerals, as a solid solution in their crystalline structure. In Castanheiro et al. (2016), sampling in a forest site, supposed without any vehicular pollution, revealed a significant amount of Fe in SEM–EDS particle analysis, which is likely due to the Fe in natural minerals. In the EDS maps we expect a contribution from natural crustal Fe-mineral and from anthropogenic Fe-bearing phases. The latter lacks Si and is generally found as tiny rounded micrometre (or less) sized crystals (Mantovani et al 2018). Si bearing phases were found only with the stoichiometry of natural minerals.

**Fig. 9:**
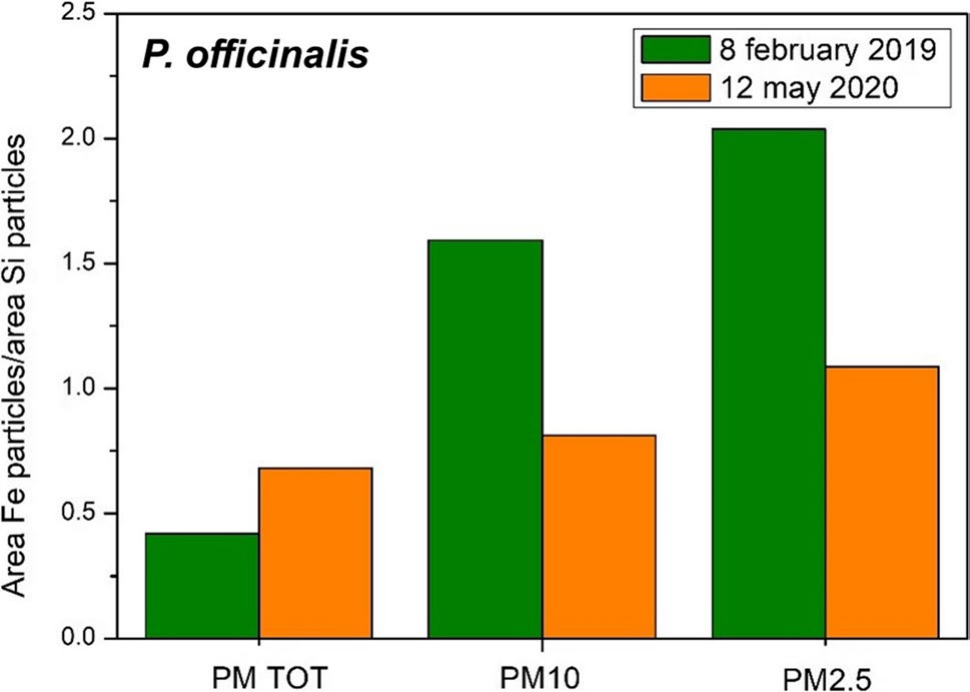
Percentage area in image mapping of Fe with area of mapping of Si

Comparing the area occupied by Fe and by Si in elemental maps, we see that Si prevails. This is related to silicates; in most of the larger grains, Fe coexists with Si as an impurity in the crystal structure, like in amphibole or in micas, but the area of Si is higher as there are several minerals that do not contain Fe, like quartz. However, if we limit the observation to particles lower in size than 10 µm, we see that the Fe and Si areas are almost the same, higher for Fe in the 2019, lower in the 2020 *P. officinalis*. In the smaller-sized grains at 2.5 µm, the Fe area prevails over Si, with a significant amount of Fe not present as an impurity in silicates. Moreover, comparing the images from 2019 and 2020 *P. officinalis*, we see a similar proportion of Fe and Si in the total particulate matter, but a higher Fe vs Si ratio as the particle size decreases in the 2019 *P. officinalis*. The suggestion is that larger grains, mostly silicates, come from a natural background, which is not affected by traffic conditions, whereas in the smaller-sized grains the higher proportion of Fe in the 2019 *P. officinalis* indicates a strong presence of smaller grains of anthropic origin, either metals or metal oxides, some of which were able to enhance a magnetic susceptibility. In Fig. 9, we observe that the Fe/Si ratio increases much more with smaller grains in the February 2019 than in the May 2020 *P. officinalis*. In the May 2020, in the *P. officinalis samples* the Fe/Si ratio does not change much in function of the grain size.

The above observations agree with the measured susceptibility, which for May 2020 are lower than any other 2019 sampling, although the value observed in *P. officinalis* and *H. helix* in 2020 still indicates a rather high magnetic mineral content.

## Conclusions

As a conclusion, we have found that (1) *P. officinalis* and *R. caesius* are significantly more efficient than *Hedera* in taking up magnetic particles. In Parma, *R. caesius* highlights a significant difference between the stations (*p* < 0.05), whereas *H. helix* does not. In the two sites in Torino, where *H. helix* and *P. officinalis* were sampled together, *P. officinalis* shows a higher magnetic susceptibility. (2) The magnetic susceptibility of samples collected around the highly trafficked urban road in Torino (CC) is significantly higher than in other locations, for both *H. helix* and *P. officinalis*. (3) In the traffic-free urban site in Torino (LPM), the magnetic susceptibility of *H. helix* is not different from that of the side of the suburban highway (T) in Parma, indicating that even a site near a pedestrian road is significantly polluted. A substantial mobility of the magnetic particles must occur: in Torino, the magnetic particles had to move about 200 m in a traffic-free area to cross the river Po to the LPM site; this could be given by the particular conformation of the Po plain: very little wind and often foggy days that do not promote air change. (4) The magnetic measurement of *P. officinalis* and *R. caesius* is not significantly different, suggesting that they have a similar bio-accumulation efficiency. (5) In *H. helix*, no significant difference was found between old and young leaves: accumulation can reach a saturation level in few days, yet to be determined. (6) The examined species look more promising to spatial monitoring, to spot sites with highest metal pollution, rather than to monitor the evolution with time, with the exception of seasonal variation. During January–February 2019, little changes in average magnetic susceptibility were observed, and only in the final sampling were an increase in the magnetic value in Parma and a decrease in Torino found. This is possibly related to the beginning of the academic period at the beginning of March in Parma, and to decreased traffic related pollution in Torino, due to the breakup of the high-pressure winter regime. (7) A prevalence of Fe-bearing metals and oxides, possibly of anthropic origin, with respect to silicates was observed in the smaller grains.

We foresee future developments in the magnetic analysis to locate pollution spots and detail the distribution of pollutants by the analysis of *P. officinalis* and/or *R. caesius*.

## Data Availability

The datasets used and/or analysed during the current study are available from the corresponding author on reasonable request.
